# Gut microbiota of two invasive fishes respond differently to temperature

**DOI:** 10.3389/fmicb.2023.1087777

**Published:** 2023-03-28

**Authors:** Lixia Zhang, Zi Yang, Fan Yang, Gege Wang, Ming Zeng, Zhongxin Zhang, Mengxiao Yang, Zhanqi Wang, Zhibing Li

**Affiliations:** ^1^Department of Ecology, College of Life Sciences, Henan Normal University, Xinxiang, China; ^2^Puyang Field Scientific Observation and Research Station for Yellow River Wetland Ecosystem and The Observation and Research Field Station of Taihang Mountain Forest Ecosystems of Henan Province, Xinxiang, China; ^3^Jigongshan National Nature Reserve, Xinyang, China; ^4^Key Laboratory of Vector Biology and Pathogen Control of Zhejiang Province, College of Life Sciences, Huzhou University, Huzhou, China

**Keywords:** gut microbiota, invasive fishes, temperature, *Cyprinus carpio*, *Micropterus salmoides*

## Abstract

Temperature variation structures the composition and diversity of gut microbiomes in ectothermic animals, key regulators of host physiology, with potential benefit to host or lead to converse results (i.e., negative). So, the significance of either effect may largely depend on the length of time exposed to extreme temperatures and how rapidly the gut microbiota can be altered by change in temperature. However, the temporal effects of temperature on gut microbiota have rarely been clarified. To understand this issue, we exposed two juvenile fishes (*Cyprinus carpio* and *Micropterus salmoides*), which both ranked among the 100 worst invasive alien species in the world, to increased environmental temperature and sampled of the gut microbiota at multiple time points after exposure so as to determine when differences in these communities become detectable. Further, how temperature affects the composition and function of microbiota was examined by comparing predicted metagenomic profiles of gut microbiota between treatment groups at the final time point of the experiment. The gut microbiota of *C. carpio* was more plastic than those of *M. salmoides*. Specifically, communities of *C. carpio* were greatly altered by increased temperature within 1 week, while communities of *M. salmoides* exhibit no significant changes. Further, we identified 10 predicted bacterial functional pathways in *C. carpio* that were temperature-dependent, while none functional pathways in *M. salmoides* was found to be temperature-dependent. Thus, the gut microbiota of *C. carpio* was more sensitive to temperature changes and their functional pathways were significantly changed after temperature treatment. These results showed the gut microbiota of the two invasive fishes differ in response to temperature change, which may indicate that they differ in colonization modes. Broadly, we have confirmed that the increased short-term fluctuations in temperatures are always expected to alter the gut microbiota of ectothermic vertebrates when facing global climate change.

## Introduction

In many cases among invertebrates and vertebrates, gut microbiota can affect host development, behavior, health, and fitness (McFall-Ngai et al., 2013; Luczynski et al., 2016; Qiao et al., 2019; Sittipo et al., 2022). Recent years, there are growing studies showing that the gut microbiota can be affected by intrinsic factors such as developmental stage and gender (Chouaia et al., 2019; Santos-Marcos et al., 2019; Hu et al., 2020; Zhang et al., 2020; Chen et al., 2021; Yoon and Kim, 2021), and also by extrinsic factors such as temperature, diet (David et al., 2014; Fontaine et al., 2018; Huyben et al., 2018; Wang et al., 2020; Kashinskaya et al., 2021), and habitat (Barelli et al., 2015; Bennett et al., 2016; Huang et al., 2018; Dulski et al., 2020; Tian et al., 2022). Compared to endotherms, ectotherms are not able to maintain relatively consistent body temperatures in the face of cold conditions (Angilletta et al., 2002). So, changes in ambient temperature could more easily alter the diversity and composition of gut microbiota of ectotherms, potentially affecting their health (Fontaine et al., 2018; Huyben et al., 2018; Fontaine and Kohl, 2020; Moeller et al., 2020; Zhu et al., 2021a; McMunn et al., 2022; Zhang et al., 2022). Further, some research has indicated that gut microbial diversity is reduced in salamanders and lizards when they experienced increases in environmental temperatures, which reduced digestive performance in former and decreased survival in latter (Bestion et al., 2017; Fontaine et al., 2018; Zhu et al., 2021a). Global climate change is occurring, therefore, the physiology in ectotherms may be influenced by an indirect effect of temperature on the gut microbial communities.

The structure and composition of gut microbiota is impacted by temperature change; however, the significance of effects may largely depend on how rapidly the gut microbiota can be altered by a change in temperature (Fontaine and Kohl, 2020). As in many taxa, longer term seasonal shifts in temperature are of the strongest drivers known to impact gut microbial communities, having been observed in both endotherms (Maurice et al., 2015; Fan et al., 2022; Marsh et al., 2022) and ectotherms (Kohl and Yahn, 2016; Ferguson et al., 2018; Li S. et al., 2022). On longer temporal scales (seasons or months), the changes of gut microbiota are mainly beneficial for host animals. Now if, supposing the gut microbial communities could be affected by temperature over shorter temporal scales (hours, days, or weeks), an increase in the frequency of extreme weather events around the world may have the severe impacts on host physiology *via* temperature change (Carey and Duddleston, 2014). Ectothermic animals should be especially suitable to investigate the hypothesis because they are more sensitive to changing temperature. However, studies about the effects of temperature change on physiology of ectotherms *via* altering gut microbiota is rarely examined (Fontaine and Kohl, 2020).

Furthermore, how gut microbiota were altered when temperature changes and how they affect metabolic functions of organisms is still unknown (Fontaine et al., 2018; Moeller et al., 2020; Sepulveda and Moeller, 2020; Tong et al., 2020). If these impacts are mediated *via* the effects of temperature on host physiology, then they may differ across host species. Indeed, a recent study has found that gut microbiota of an ectothermic species was changed more significantly than those of counterpart by increased temperature (Fontaine and Kohl, 2020). It has been posed that altered microbial communities in response to environmental stress have beneficial impact on host physiology, which would increase their ability to adapt to changing conditions (Alberdi et al., 2016; Moeller et al., 2020). Under such circumstances, species harboring more plastic gut microbiota are expected to exhibit higher phenotypic plasticity when navigating novel environments, which was often occurred in invasive species (Davidson et al., 2011; Xu et al., 2019). Colonization ability is one of the most important characteristics of species which can facilitate habitat expansion. Thus, understanding how temperature affects gut microbiota in multiple host species that with well-developed colonization abilities can shed light on the invasive organism’s capacity for range expansion.

Many studies have detected that alternative environmental temperature can influence gut microbial communities of ectotherms (Kohl and Yahn, 2016; Bestion et al., 2017; Horlick et al., 2020; Li et al., 2020; Zhu et al., 2021a), however, few studies have investigated how quickly temperature can alter ectothermic gut microbial communities (Fontaine and Kohl, 2020). To understand this issue, we exposed juvenile fish to increased environmental temperature and sampled of the gut microbiota at multiple time points after exposure so as to determine when differences in these communities first become detectable. In addition, how temperature affects the composition and function of microbiota was examined by comparing predicted metagenomic profiles of gut microbiota between treatment groups at the final time point of our experiment. For gaining useful knowledge on how host biology affects this pattern, the experiment was conducted in juveniles of two freshwater fishes, the common carp (*Cyprinus carpio*) and the largemouth bass (*Micropterus salmoides*). The carp has no distinct stomach and was omnivorous in its feeding habits (Rahman et al., 2008; Dadebo et al., 2015). The bass is carnivorous inhabits and feeds on various zooplankton, arthropods, and even larger prey such as other fish, amphibians, and birds (Cochran and Adelman, 1982; Brown et al., 2009). Its large stomach extend at the start of the intestine and help in nutrient absorption (Reifel and Travill, 1979; Buddington and Diamond, 1987). The intestine of the bass is short and thick walled, so the digestion rate of the bass is much greater than other herbivorous or omnivorous fishes (Hunt, 1960; Reifel and Travill, 1979; Day et al., 2014). Growing numbers of studies suggest that these species provide us an ideal model system to test our questions because fish gut microbiota is not only important for normal physiology of the host (Larsen et al., 2014; Ghanbari et al., 2015; Liu et al., 2016; Wang et al., 2018; Liu Q. et al., 2021; Liu Y. et al., 2021) but is also temperature dependent (Larios-Soriano et al., 2021; Liu et al., 2022). The both fish species share ecological similarities, and they both ranked among the 100 worst invasive alien species in the world (Lowe et al., 2000). The hypothesis suggested that the plasticity of the gut microbiota can be an essential factor determining phenomic plasticity of vertebrates, and that it can play a vital role when vertebrates acclimate and adapt to fast environmental variation (Alberdi et al., 2016). So, we predicted that gut microbiota of the two fishes would both exhibit rapid plasticity, potentially contributing to their adaptation to new environmental conditions (e.g., climate change).

## Materials and methods

### Animals and samples

Two juvenile fishes used in this study were procured from a freshwater fish breeding base in Zhengzhou, China. The initial mean body weight of common carp and largemouth bass was 26.16 ± 4.75 g and 23.68 ± 4.51 g, respectively. The experiment was conducted in the climate-controlled environmental chamber at the Henan Normal University. Prior to the experiment, 12 individuals from each fish species were housed in small polycarbonate tank (common carp: 96 fish and 8 tanks; largemouth bass: 96 fish and 8 tanks) with 15 L of dechlorinated tap water (pH: 7.0 ± 0.5; dissolved oxygen: 6.60 ± 0.1 mg/L; photoperiod: 12-h light:12-h dark). The water temperature was kept constant at 24°C with aquarium heaters. These fishes were reared on artificial diets and acclimatized for a period of 7 days. For each tank, the artificial diet was sterilized by autoclaving (115°C, 30 min) and given daily at 2% of the fish biomass (feeding time: 8:00). In addition, one-third of water was replaced every day by dechlorinated tap water with the same temperature.

After being acclimated for 7 days, the water temperature in four tanks of each species were increased from 24 to 29°C in 75 min (1°C/15 min) using aquarium heaters (Warm group). The other four tanks remained at 24°C (Cool group). Our lower temperature limit is closer to the most common temperature experienced by aquatic organism during summer in temperate zone (Brattstrom, 1963; Fontaine and Kohl, 2020). Our higher temperature limit is closer to the maximum weekly mean temperatures for largemouth bass and common carp (Eaton et al., 1995). So, the two temperatures are ecologically relevant to our study species. In addition, studies have shown that the minor temperature differences (e.g., 5°C) can significantly affect gut microbial communities in animals (Fontaine et al., 2018; Fontaine and Kohl, 2020). The temperature of each tank was monitor constantly and temperature readings were remained within ± 0.5°C of target. One day (24 h), 3 days (72 h), and 7 days (168 h) after temperature increase, four specimens of both fish species were randomly chosen from each tank and euthanized by MS-222. The both fishes were sampled after 1 h of feeding. Each individual was dissected and the whole gut contents (only intestines) were collected. The content from two individuals was mixed so as to decrease sample heterogeneity. Then, the gut samples were immediately frozen in liquid nitrogen. In order to remove the potential external contaminants, all samplings in the experiment were carried out in super clean workbench. In addition, all dissection instruments were washed with 100% EtOH between individuals and soaked in 20% bleach solution for at least 5 min before dissection (Linville and Wells, 2002).

### Molecular analyses and sequence processing

The DNA extraction from microorganisms was done by using E.Z.N.A.^®^ soil DNA Kit (Omega Bio-Tek, Norcross, GA, USA). The PCR product of 16S rRNA was generated with the primers 338F (5′-ACTCCTACGGGAGGCAGCAG-3′) and 806R (5′-GGACTACHVGGGTWTCTAAT-3′). Detailed description of PCR amplification was reported in Supplementary Section 1.1. PCR amplicons were purified both by gel isolation and DNA purification kit (Axygen Biosciences, Union City, CA, USA). The purified products were quantified by QuantiFluor™-ST (Promega, Madison, WI, USA) and pooled at equimolar concentrations. Then, the V3 and V4 regions of the 16S ribosomal RNA gene were sequenced on Illumina MiSeq platform. Detailed description of sequence was provided in Supplementary Section 1.2.

### Biodiversity analysis

The diversity of gut bacteria of both species from warm and cool group was analyzed by Illumina MiSeq sequencing. The MOTHUR software was used for alpha-diversity analyses, including number of observed OTUs (operational taxonomic units), Shannon diversity index, and Faith’s phylogenetic diversity. Rarefaction curves were created using USEARCH (version 7), which can used to understand the depth of sampling of a community compared with its total diversity. Beta diversity measurements was performed through Clustering analysis by principal coordinate analysis (PCoA), which illustrates the differences in species composition between assemblages or regions. The composition and abundance diagrams of gut microbial communities at phylum and genus levels were analyzed by QIIME software (Version 1.9.1). The differences between warm and cool groups were made by Welch’s *t*-test. The criterion of statistical significance was *P* < 0.05.

### Statistical analysis

#### Comparison of gut microbiota between host species

To test for differences in alpha diversity by species, the linear mixed models (LMMs) were performed in R (version 3.3.1), using time and temperature as the independent variable. To evaluate for differences in gut microbiota composition by host species, PERMANOVA (permutational multivariate analysis of variance) tests were applied to Bray–Curtis dissimilarity matrix, unweighted UniFrac and weighted UniFrac distance matrices with 999 permutations, which were performed in QIIME. False discovery rate (FDR) corrected *p*-values were reported to account for multiple comparisons. PCoA plots were generated in R, which can visualize the data.

To determine which bacterial phyla and genera had mean relative abundances that differed significantly between host species, *t*-tests in JMP (version 16) were performed with the relative abundance of each taxa as response variables. For this analysis, the relative abundance data were normalized using an arcsine square root transformation (Shchipkova et al., 2010). We used the response screening function in JMP to provide FDR corrected *P*-values. To reduce the influence of very rare taxa, only those present at >1% relative abundance in samples of both host species were included in the statistical analyses (Chen et al., 2018; Fontaine and Kohl, 2020).

#### Effects of experimental treatments on the gut microbiota within each species

To identify effects of temperature, time and their interaction on the gut microbiota alpha diversity of each species, LMMs were constructed in R. To explore how temperature and time impacted gut microbiota of each species, the analyses were performed using QIIME with PERMANOVA, based on Bray–Curtis distance matrices. Each model used 999 permutations and FDR corrected *p*-values were reported to account for multiple comparisons. In addition, PERMANOVA was performed using the Adonis function in the vegan package in R, which were employed to examine at what time point a significant change occurred in high temperature stress. Using each of the three distance matrices (Bray–Curtis dissimilarity matrix, unweighted UniFrac and weighted UniFrac distance matrices), pairwise PERMANOVA were used to test significant differences between the two levels of temperature within each time point. By employing R, the results of these analyses were presented in the form of PCoA plots.

We further used JMP to explore which dominant bacterial phyla or genera that were influenced by temperature, time, or the interaction of the two variables using the Response Screening function to conduct multiple ANCOVAs and correct *P*-values with the FDR correction. The ANCOVA models were performed with the relative abundance of each taxa were used as response variables, while temperature, time, or the interaction of the two variables used as predictor variables (Benjamini and Hochberg, 1995). Prior to analysis, the relative abundance data were used arcsine-squareroot transformation to normalize distributions (Shchipkova et al., 2010). Again, only those present at >1% relative abundance in samples of both host species were included in the statistical analyses.

Lastly, 16S rRNA sequencing at the end of the experiment (168 h, 7 days) and PICRUSt2 (Phylogenetic Investigation of Communities by Reconstruction of Unobserved States) software were employed to investigate how increased temperature could affect the function of gut microbial communities. To obtain compatibility data for PICRUSt2 software, separate OTU tables for each host species were created by using the closed-reference OTU clustering which was done at 99% similarity level against the GreenGenes database (v13_8) (McDonald et al., 2012). Then, we put OTU table into PICRUSt2 for metagenome predication by using the KEGG (Kyoto Encyclopedia of Genes and Genomes) database. Comparison among pathways which had relative frequencies that were affected by temperature were conducted using Welch’s two-sample *t*-test in R by removing unclassified reads from analysis. Here again, the FDR method were used to correct for multiple comparisons.

## Results

### Comparison of gut microbiota between host species

From the common carp intestinal samples, a total of 2,646,278 high-quality sequences (mean sequences for each sample: 57,528 ± 8,225) were obtained from all samples. These high-quality sequences were clustered into OTUs at the 97% similarity level, generating 543 OTUs. In addition, we obtained a total of 2,247,532 high-quality sequences (mean sequences for each sample: 49,945 ± 6,656) and 596 OTUs from largemouth bass intestinal samples. The rarefaction curves showed a sufficient sequencing depth (Supplementary Figures 1A, B). After removing rare taxa (abundance below 1% threshold per sample), 11 phyla for both fishes, and 63 and 56 genera in carps and basses were retained, respectively.

At the end of the experiment, results of LMM analysis revealed that the alpha diversity of microbiota (i.e., Number of observed OTUs; Shannon diversity index; Faith’s phylogenetic diversity) was higher in largemouth bass than in common carp when accounting for time and temperature (all *p* < 0.05). Further, we detected that microbial community structure differed significantly between the two species, regardless of temperature or time (Supplementary Figure 2; *P* = 0.001, PERMANOVA of Bray–Curtis dissimilarities). When accounting for temperature and time, we founded that the mean relative abundance of five dominant bacterial phyla differ greatly between the two fish species (Proteobacteria, Firmicutes, Actinobacteriota, Verrucomicrobiota, Bacteroidota, *t*-test, all *p* < 0.05; Supplementary Table 1). Specifically, four phyla were more abundant in common carp whereas another one phyla were more abundant in largemouth bass. At the genera level, the relative abundances of 40 bacterial genera differed between the two fish species (*t*-test, all *p* < 0.05; Supplementary Table 1). Specifically, 32 genera were more abundant in common carp whereas another 8 genera were more abundant in largemouth bass.

### Effects of experimental treatments on the gut microbiota within each species

There are no significant effects of temperature and the interaction of temperature and time on alpha diversity of gut bacteria in common carp (LMMs, all *p* > 0.05), however, the significant effect of time on OTUs, Shannon diversity index and Faith’s phylogenetic diversity was detected in carps gut bacteria (Supplementary Figures 3A1–A3; LMMs, all *p* < 0.001). The temperature and the interaction of temperature and time had no effect on all three metric of alpha diversity of gut bacteria in largemouth bass (LMMs, all *p* > 0.05), however, the significant effect of time on OTUs and Faith’s phylogenetic diversity was detected in the bass (Supplementary Figures 3B1–B3; LMMs, *p* < 0.05).

In common carp, our results showed that the temperature and time had a major impact on the bacterial composition of the gut microbiome based on Bray–Curtis dissimilarity between samples (Supplementary Table 2; PERMANOVA, all FDR *p* < 0.05). For basses, however, we found that their gut microbiome was only significantly affected by temperature based on Bray–Curtis dissimilarity (Supplementary Table 2; PERMANOVA, all FDR *p* < 0.05).

**FIGURE 1:**
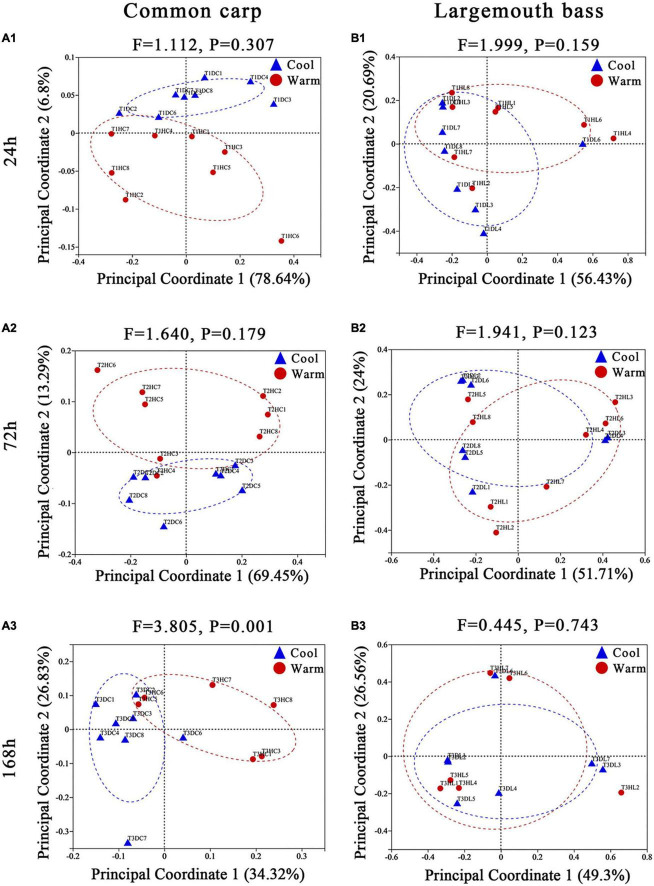
Principal coordinate analyses plots of common carp and largemouth bass gut microbiota at the 24 h **(A1,B1)**, 72 h **(A2,B2)**, and 168 h **(A3,B3)** experimental time points, based on weighted UniFrac distance across samples. Points are colored by temperature treatment (red = warm; blue = cool), and ellipses represent the 95% confidence interval of that treatment group. On each plot, the results of PERMANOVA models assessing the temperature effects on gut microbial community composition at that time point are displayed, including the *F* statistic from the model, and the FDR corrected *p*-values (*q*-value). Percentages on the axes of PCoA plots indicate the proportion of variation explained by that axis.

Based on Bray–Curtis and weighted UniFrac distances between samples, the pairwise models comparing gut microbiome composition by temperature within each of the three time points showed that gut microbiome of carps under the two temperature treatments were became significantly different at the third time points of the experiment (168 h, 7 days) (Supplementary Table 3; Figures 1A1–A3; Supplementary Figures 4A1–A3; PERMANOVA, all FDR *p* < 0.01). In addition, microbiota variation in carps was detected between the two temperature treatments at any time point of the experiment when using unweighted distance (Supplementary Table 3; Supplementary Figures 5A1–A3; PERMANOVA, FDR *p* < 0.01 at every time point). For largemouth bass, microbiota variation by temperature were only observed at the second time points (72 h, 3 days) during the experiment period by using the Bray–Curtis and unweighted UniFrac dissimilarity (Supplementary Table 3 and Supplementary Figures 4B1–B3, 5B1–B3; PERMANOVA, all FDR *p* < 0.05). But, the analysis based on weighted UniFrac distance revealed no significant effect of temperature on gut microbiome across all time points (Supplementary Table 3; Figures 1B1–B3; PERMANOVA, FDR *p* > 0.05 at every time point).

**FIGURE 2:**
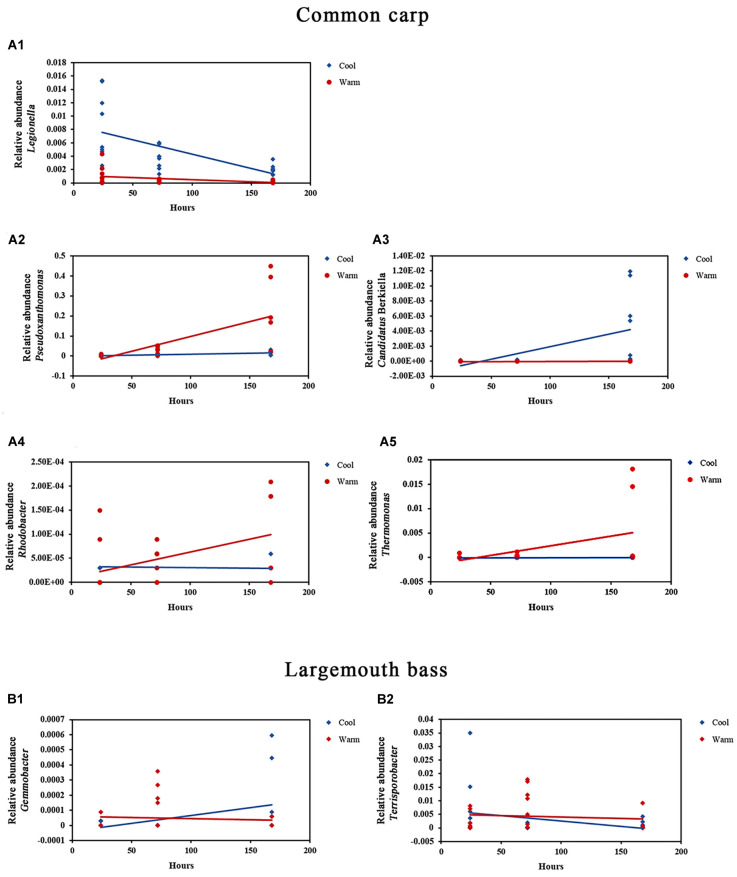
Relative abundances of bacterial genera in the gut microbial communities of common carp and largemouth bass that were significantly affected by the interaction of temperature and time. **(A1–A5)** Relative abundance of *Legionella*, *Pseudoxanthomonas*, *Candidatus* Berkiella, *Rhodobacter*, and *Thermomonas* in common carp gut microbial communities in warm and cool treatments over time, respectively (all FDR *p* < 0.05). **(B1,B2)** Relative abundance of *Gemmobacter* and *Terrisporobacter* in largemouth bass gut microbial communities in warm and cool treatments over time, respectively (all FDR *p* < 0.05).

In gut microbiome of common carps, 3 phylum and 13 genera with relative abundances that were influenced by temperature, or by interaction of temperature and time factors (Table 1 and Figures 2A1–A5; ANCOVA, FDR *p* < 0.05 for all taxa). In addition, there are 6 phyla and 29 genera of the host fish with higher relative abundances that were only affected by time (Supplementary Table 4; ANCOVA, FDR *p* < 0.05 for all taxa). In gut microbiome of largemouth bass, none phylum and 6 genera with relative abundances that were influenced by temperature, or by interaction of temperature and time factors (Table 1 and Figures 2B1, B2; ANCOVA, FDR *p* < 0.05 for all taxa). Further, there are one phyla and five genera of the host fish with higher relative abundances that were only affected by time (Supplementary Table 4; ANCOVA, FDR *p* < 0.05 for all taxa).

**TABLE 1:** Bacterial phyla and genera in common carp and largemouth bass gut microbiota which had relative abundances that were significantly affected by temperature or the interaction of temperature and time.

		Temp	Temp × Time	*F* statistic	*q*-value
**Common carp**	**Phylum**				
	Actinobacteriota	C		7.811	<0.05
	Verrucomicrobiota	C		4.833	<0.01
	Planctomycetota	C		8.932	<0.05
	**Genera**				
	*Legionella*		↓ (C)	24.360	<0.001
	*Pseudoxanthomonas*	W	↑ (W)	15.786	<0.001
	*Pseudoxanthobacter*	C		12.348	<0.01
	*Candidatus* Berkiella		↑ (C)	11.237	<0.001
	*alphaI* cluster	C		10.277	<0.001
	*Rhodobacter*		↑ (W)	7.695	<0.01
	*Plesiomonas*	W		5.756	<0.05
	*Gemmobacter*	W		5.500	<0.05
	*Aurantimicrobium*	C		4.906	<0.05
	*Microbacterium*	W		4.318	<0.05
	*Thermomonas*		↑ (W)	4.205	<0.05
	ZOR0006	W		4.058	<0.05
	*Deinococcus*	C		3.704	<0.05
**Largemouth bass**	**Genera**				
	*Plesiomonas*	W		4.352	<0.001
	*Peptostreptococcaceae*	C		3.666	<0.01
	*Gemmobacter*		↑ (C)	3.176	<0.05
	*Edwardsiella*	W		4.725	<0.01
	*Epulopiscium*	W		3.340	<0.05
	*Terrisporobacter*		↓ (C)	2.553	<0.05

The symbol in the temperature column (temp) indicates temperature effects, (W) indicates greater abundance of this taxa in warm temperatures, (C) indicates greater abundance of this taxa in cool temperatures. The symbol in the temp **×** time column indicates the interaction of temperature and pressure effects, arrows indicate the direction of the relationship of that taxa’s abundance with time (increasing or decreasing), and (W) or (C) indicates that change with time occurred in warm or cool temperatures, respectively. Significance was determined with ANCOVA models and the *F* statistic for each variable is presented, along with FDR corrected *p*-values (*q*-values). Taxa are ordered by their effect size.

**FIGURE 3:**
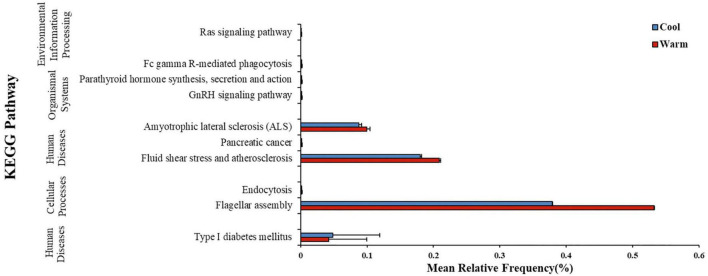
Mean ± SD relative frequency (%) of KEGG pathways that differed in relative frequency across two temperature treatment groups after 168 h (7 days) of exposure to the temperature treatment in common carp gut bacterial communities. KEGG pathways were identified from predicted metagenomic profiles created using the PICRUSt2 program and significance by temperature groups was determined using a Welch’s two-sample *t*-test, with FDR corrected *p*-values.

In gut microbiome of common carps, PICRUSt2 analysis suggested that the warm temperature group at the final time point had 9 KEGG pathways which was significantly higher in relative frequency than those in the low temperature group at the same time (Figure 3; Welch’s two-sample *t*-test, FDR *p* < 0.05 for all pathways). In gut microbiome of largemouth bass, however, PICRUSt2 analysis suggested that the high temperature group at the final time point had none pathways which was higher or lower in relative frequency than those in the low temperature group at the same time (Welch’s two-sample *t*-test, FDR *p* > 0.05 for all pathways). Thus, the influence of temperature on the predicted metagenome of carps was relatively strong than that on those of largemouth bass.

## Discussion

In nature, the animals that the global climate change will affect at most are ectotherms partly because their performance were significantly affected by daily temperature fluctuations (Paaijmans et al., 2013; Pincebourde and Casas, 2015; Rozen-Rechels et al., 2019). Therefore, studies on short-term changes in environmental temperature in ectotherm physiology are urgently needed, including microbial communities that play important roles in host health and respond relatively quickly to changes in the environmental conditions (McFall-Ngai et al., 2013; Horlick et al., 2020; Li J. et al., 2022). In the present study, we examined how temporal temperature variation affects gut microbial community composition in two fish species (the common carp and largemouth bass) by an increase in environmental temperature at three time points (ranging from 24 to 168 h). Our results demonstrate that the composition of gut microbiota in ectotherms can be influenced by short-term changes in temperature, though these effects are host-species specific.

We showed that the gut microbiota of common carp was more plastic than that of largemouth bass and was more sensitive to temperature changes. Specifically, the composition of gut microbiota was altered by temperature more rapidly in carps than in basses. It is known that the two fish species both ranked among the 100 worst invasive alien species in the world (Lowe et al., 2000). For common carp, it is widely distributed in natural water bodies and is being considered a serious threat to native aquatic communities and ecosystems in Canada, Australia, and USA (Zambrano et al., 2006; Chapman and Hoff, 2011; Weber and Brown, 2011; Forsyth et al., 2013; Bajer and Sorensen, 2015; Bajer et al., 2016). For largemouth bass, studies have showed that in areas where it is present, native prey species populations are often decimated and other native fish species are absent (Weyl et al., 2010; Ellender et al., 2011). There is research that further corroborates these reports (Alexander et al., 2014). Compared to natives, the successful invasive species are expected to have wider adaptability to different environments, for native species are generally less phenotypically plastic than invasive species (Davidson et al., 2011). As an example, invasive lionfish (*Pterois* sp.) has a relatively large change in CTmax with temperature, presumably enabling the lionfish to cope well with environmental temperature increase and possibly facilitating their invasive success across a wide range of habitats (Barker et al., 2018). Further, a recently study has reported that there is a high level of thermal resilience in round goby, which is an invasive fish around the world (Christensen et al., 2021). The high levels of phenotypic buffering of metabolism could benefit invasive species through enhanced thermal resilience, when compared to surrounding native species. It has been proposed that gut microbiota can show plasticity in response to environmental change, and thus aid host adaption to survive under novel and stressful conditions (Alberdi et al., 2016; Khakisahneh et al., 2020; Zhu et al., 2021b). However, our results showed that the gut microbiota of the two invasive fish species differ in response to temperature change, which may indicate that they differ in colonization modes.

The gut microbiota of common carp not only exhibit plasticity in response to temperature changes, but also generates plasticity in their function. In carps significantly more pathways were affected by increased temperature. Among these pathways, one of which is known to be associated with endocytosis, one of which is known to be associated with phagocytosis. Both pathways were increased in frequency when exposure to increased temperature. In addition, a significant decrease in endocrine and metabolic disease metabolism pathway at high temperatures was detected in carps. These results may indicate that gut microbiota can promotes host resistance to high-temperature stress by stimulating its endocytosis and phagocytosis-related pathway in carps, thus potentially enhancing immune activation in this species. This finding is inconsistent with results from other studies that found 26 pathways of energy, vitamins, and protein metabolism were significantly increased in frequency when exposure to increased temperature in invasive bullfrog tadpoles (Fontaine and Kohl, 2020). In contrast, in bass, none pathways were changed in frequency when exposure to increased temperature. This differs in response to high-temperature stress suggests that the adaptive response to environmental shifts differ between the two invasive fish species.

Facing global climate change, irrespective of invasiveness, finding about how temperature affects microbiota composition and activity in ectotherm can have implications for animal physiology and health. Some studies have demonstrated that seasonal temperature changes can lead to gut microbiota changes in composition and abundance of microbiota in mammals (Carey et al., 2012; Maurice et al., 2015; Liu et al., 2019; Xiao et al., 2019). In addition, multiple studies have shown that gut microbiota of ectothermic animals can be modified by long-term (weeks to years) warm temperature exposure (Kohl and Yahn, 2016; Bestion et al., 2017; Fontaine et al., 2018; Huyben et al., 2018; Dulski et al., 2020; Huang and Liao, 2021; Kashinskaya et al., 2021). Our study found that the composition of an individual’s gut microbiota can be highly plastic in response to changing temperature on rapid time scales (from hours to days). This finding could be confirmed by previous studies (Sun et al., 2017; Fontaine et al., 2018; Fontaine and Kohl, 2020). Due to the climate change, the frequency and magnitude of short-term extreme weather events such as weeklong heat waves are predicted to be common in many parts of the world. Therefore, the gut microbiota of ectotherms was expected to become less stable. Under this scenario, healthy of species would be impaired if the quantities of some important bacterial taxa were suppressed at the class and genus levels. Indeed, studies have demonstrated that the increased temperature significantly affects digestive performance of a salamander (Fontaine et al., 2018) and altered energy metabolism of gut microbiota in a lizard (Bestion et al., 2017). In addition, the relative abundance of some pathogenic taxa were significantly higher in high temperature group (Fontaine et al., 2018). Our study demonstrated that the relative abundance of genus *Plesiomonas* was significantly increased in both fish samples from the warm temperature treatment group, which has been implicated as a cause of occasional gastrointestinal disease (Clark and Janda, 1991). Additionally, our study demonstrated that the relative abundance of the genus *Edwardsiella* and *Epulopiscium* were higher in bass with warm temperature conditions. The former genus has been demonstrated to be pathogenic for humans (Michael and Abbott, 1993) and the later genus can influence digestive enzyme activity of an herbivorous surgeonfish (Pollak and Montgomery, 1994). Although members of the same bacterial genus are pathogenic for human and animals, there are some species that are considered commensal of the genital tract in fish. Thus, the impact of one genus on animal health is complex to assess. To discover how gut microbiota altered by high temperature impact host physiology, further studies about fecal microbiota transplants are required.

By nowadays, the effects of changing temperature on ectothermic gut microbial community composition and function are mainly derived from studies in one species (Kohl and Yahn, 2016; Bestion et al., 2017; Fontaine et al., 2018; Huyben et al., 2018). Therefore, the question about microbial growth was directly affected by temperature or mediated through temperature-induced changes in host physiology remained unclarified. We found that the gut microbiota composition of host fish species differ in response when the temperature was elevated, which implying these changes are at least partly host-mediated. Many aspects of ectotherm physiology can be affected by environmental temperature (Angilletta et al., 2002), and thus microbial community structure can be known to influence certain physical functions. Indeed, many studies have shown that the immunity of fish could be affected by temperature (Collazos et al., 1994; Alcorn et al., 2002; Nikoskelainen et al., 2004; Abram et al., 2017; Rem, 2019) and the immunity response of fish can be regulated by intestinal microbiota (Xiong et al., 2020; Sun et al., 2021). Additionally, it has been discovered that gut microbiota has effects on behavior, growth and digestive system of fish (Bairagi et al., 2002; Ray et al., 2012; Butt and Volkoff, 2019). The dietary components of host have a greater effect on the gut microbial community composition of aquatic animals, which in turn influences all of the above factors in fish (Baldo et al., 2015; Zhou et al., 2018; Kashinskaya et al., 2021). In fact, the same physical traits response to thermal stress differs among ectothermic animals (Simon et al., 2015; Fontaine and Kohl, 2020). So, when and how gut microbial communities were affected by temperature change could be partly determined by how physical disgust sensitivity of host to temperature change.

Although temperature exposure influences composition of the gut microbial community, these changes are at least somewhat influenced by the initial gut microbial community of specific host. Namely, if one host contains more sensitive microbial communities than those of another host, its microbiome may be more plastic in response to temperature changes. At the begin of manipulations, the gut microbiome of both fish was not compared. At the end of manipulations, a distinct bacterial community of carps from basses were detected, regardless of temperature or time. The *Pseudoxanthomonas* was enriched compared to low-temperature group in carps. With thermal assistance, this genus can be enriched for degrading lignocellulose (Ma et al., 2021). In basses, we found the most relatively abundant phyla were Firmicutes, Fusobacteria, Bacteroidetes, and Proteobacteria, which were consistent with previous research (Yu et al., 2021). In this fish, the *Epulopiscium*, was enriched compared to low-temperature group. The enriched genus was also detected in gut microbiome of milkfish that experienced thermal stress (Hassenrück et al., 2020), which has been identified as a prominent member of the gut microbiome in fish exhibiting a symbiotic relationship with their host (Montgomery and Pollak, 1988; Boonanuntanasarn et al., 2018).

Irrespective of temperature, the time effect on community composition of gut microbiota were noticed in both fish species. The differences in diet and environmental factors such as water temperature between controlled experimental conditions and captive-natural environments can potentially alter the associated microbiota of hosts, including fish (Dhanasiri et al., 2011; Tan et al., 2019). Thus, these changes could partly explain the effects of time observed by this experiment. In addition, the density of fish in tanks consistently decreased as individuals were removed from each tank at each time point, which could decrease levels of competition and potentially increase food intake (Zhou et al., 2011). Therefore, both the above-mentioned reasons could alter gut microbiota of aquatic ectotherms.

Under the extreme climate change scenario, the increased short-term fluctuations in temperatures are expected to significantly affect the physical performance of ectotherms (Paaijmans et al., 2013). Our study has confirmed that the composition of gut microbiota can be influenced by these changes in temperature, which play an important physical role in host immunity and metabolism. Building a better understanding of how past, current and future weather events impact gut microbiota can help improve our assessments of physiology and health of wildlife populations on a large time scale. However, the effect of temperature on ectotherm gut microbiota was highly specific for a given host, which potentially helps the invasive species to invade and expand in new habitats. Thus, studies examining the degree to which these shifts differentially impact physiology and fitness of species should be considered a future research priority.

## Data availability statement

The datasets presented in this study can be found in online repositories. The names of the repository/repositories and accession number(s) can be found below: https://www.ncbi.nlm.nih.gov/, PRJNA903526 and PRJNA903555.

## Ethics statement

This animal study was reviewed and approved by the Ethics Committee of Henan Normal University.

## Author contributions

LZ and ZY: conceptualization, visualization, writing—original draft, and supervision. FY, GW, MZ, ZZ, and MY: data curation, investigation, formal analysis, methodology, and software. ZW and ZL: methodology and writing—review and editing. All authors contributed to the article and approved the submitted version.

## Supplementary material

Supplementary Figure 1 Rarefaction curves of intestinal microbial samples based on Illumina MiSeq sequencing. **(A)** Common carp. **(B)** Largemouth bass.

Supplementary Figure 2 Principal coordinate analysis plot displaying gut microbiota of common carp and largemouth bass, based on Bray–Curtis dissimilarity across samples. The plot includes all samples, from each temperature and time point. Percentages on PCoA axes represent the proportion of variation explained by each axis. Two groups are significantly different from one another (PERMANOVA, FDR *P* = 0.001).

Supplementary Figure 3 Alpha diversity of gut microbiota of fishes in two temperature treatments at three time points. The center bolded line represents the median, the length of the box represents the IQR, and the whiskers extend to 1.5xIQR. Points beyond this value are plotted individually. Common carp **(A1–A3)**, Shannon diversity, OUTs and PD index of common carp was significantly impacted by time (GLMM, *p* < 0.001), but not by temperature (GLMM, *p* > 0.05). Largemouth bass **(B1–B3)**, OUTs and PD index of largemouth bass was significantly impacted by time (GLMM, *p* < 0.05), but not by temperature (GLMM, *p* > 0.05).

Supplementary Figure 4 Principal coordinate analyses plots of common carp and largemouth bass gut microbiota at the 24 h **(A1,B1)**, 72 h **(A2,B2)**, and 168 h **(A3,B3)** experimental time points, based on Bray–Curtis dissimilarity across samples. Points are colored by temperature treatment (red = warm; blue = cool), and ellipses represent the 95% confidence interval of that treatment group. On each plot, the results of PERMANOVA models assessing the temperature effects on gut microbial community composition at that time point are displayed, including the *F* statistic from the model, and the FDR corrected *p*-values (*q*-value). Percentages on the axes of PCoA plots indicate the proportion of variation explained by that axis.

Supplementary Figure 5 Principal coordinate analyses plots of common carp and largemouth bass gut microbiota at the 24 h **(A1,B1)**, 72 h **(A2,B2)**, and 168 h **(A3,B3)** experimental time points, based on unweighted UniFrac distance across samples. Points are colored by temperature treatment (red = warm; blue = cool), and ellipses represent the 95% confidence interval of that treatment group. On each plot, the results of PERMANOVA models assessing the temperature effects on gut microbial community composition at that time point are displayed, including the *F* statistic from the model, and the FDR corrected *p*-values (*q*-value). Percentages on the axes of PCoA plots indicate the proportion of variation explained by that axis.
