# Supplementary material for: Gut microbiota of two invasive fishes respond differently to temperature
Source: Front Microbiol. 2023 Mar 28;14:1087777. doi: 10.3389/fmicb.2023.1087777 (PMC10088563; doi:10.3389/fmicb.2023.1087777)
Supplement: Supplementary file 1 [file Data_Sheet_1.docx]

**SUPPORTING INFORMATION**

**Supplemental materials and methods**

**1.1 PCR amplification**

All PCR reactions were conducted in triplicate using a total volume of 20 μL reaction system containing 4 μL 5 × FastPfu Buffer, 2 μL 2.5 mM dNTPs, 0.8 μL each primer (5 μM), 0.4 μL FastPfu Polymerase, and 10 ng template DNA. Thermal cycling was consisted of initial denaturation at 95°C for 3 min, followed by 27 cycles of denaturation at 95°C for 30 s, annealing at 55 °C for 30 s, and elongation at 72 °C for 30 s. Eventually, the PCR system was held at 72 °C for 10 min.

**1.2 Sequence processing**

Raw 16S rRNA data were demultiplexed, quality filtered using the Trimmomatic and FLASH software with the following three criteria. Firstly, low quality reads with scores < 20 or with a read length < 50 bp were filtered out. Secondly, barcodes were matched, while ambiguous bases and unmatched barcodes were removed. Only overlaps sequences longer than 10 bp were assembled according to their overlap sequences. Reads that could not be assembled were discarded. Thirdly, sequences of each sample were separated according to barcodes (exactly matching) and Primers (allowing 2 nucleotide mismatching), and reads containing ambiguous bases were removed. The operational taxonomic units (OTUs) were clustered with 97% similarity cutoff using UPARSE (version 7.1 <http://drive5.com/uparse/>) and chimeric sequences were identified and removed using UCHIME. The taxonomy of each 16S rRNA gene sequence was analyzed by RDP Classifier algorithm (http://rdp.cme.msu.edu/) against the Silva (SSU115) 16S rRNA database using a confidence threshold of 70% (Amato et al., 2013).
