# Supplementary material for: Gut microbiota of two invasive fishes respond differently to temperature
Source: Front Microbiol. 2023 Mar 28;14:1087777. doi: 10.3389/fmicb.2023.1087777 (PMC10088563; doi:10.3389/fmicb.2023.1087777)
Supplement: Supplementary file 2 [file Table_1.docx]

**TABLE S1** Relative abundance (%) ± SD of bacterial phylum and genera in the gut microbiota of carps and basses that were significantly different between common carps and Largemouth basses, based on *t*-tests. Taxa are ordered based on in which species they are more abundant, and subsequently by the degree of the effect size (F statistic). P-values are FDR corrected (q-value) to control for multiple comparisons.

| **Phylum** | **Relative Abundance** | | **F-tatistic** | **q value** |
| --- | --- | --- | --- | --- |
|  | **Common carp** | **Largemouth bass** |  |  |
| Verrucomicrobiota | 0.13 ± 0.08 | 0.01 ± 0.07 | 97.433 | ＜0.01 |
| Actinobacteriota | 0.40 ± 0.15 | 0.10 ± 0.13 | 96.578 | ＜0.01 |
| Proteobacteria | 0.71 ± 0.22 | 0.41 ± 0.36 | 23.601 | ＜0.01 |
| Bacteroidota | 0.08 ± 0.07 | 0.04 ± 0.09 | 5.471 | ＜0.05 |
| Firmicutes | 0.08 ± 0.05 | 0.61 ± 0.40 | 77.229 | ＜0.01 |
| **Genara** |  |  |  |  |
| *Roseomonas* | 0.16 ± 0.06 | Not observed | 275.775 | ＜0.01 |
| *Bosea* | 0.09 ± 0.03 | 0.01 ± 0.01 | 273.425 | ＜0.01 |
| *Microbacterium* | 0.17 ± 0.07 | 0.01 ± 0.02 | 214.295 | ＜0.01 |
| *Defluviimonas* | 0.15 ± 0.07 | Not observed | 210.695 | ＜0.01 |
| *Kaistia* | 0.09 ± 0.04 | Not observed | 202.250 | ＜0.01 |
| *Rhodobacter* | 0.13 ± 0.06 | Not observed | 199.814 | ＜0.01 |
| *Aminobacter* | 0.08 ± 0.04 | Not observed | 160.405 | ＜0.01 |
| *Shinella* | 0.06 ± 0.03 | Not observed | 137.936 | ＜0.01 |
| *Gemmobacter* | 0.19 ± 0.11 | Not observed | 131.669 | ＜0.01 |
| *IMCC26207* | 0.14 ± 0.08 | 0.01 ± 0.02 | 126.509 | ＜0.01 |
| *Phreatobacter* | 0.04 ± 0.02 | 0.01 ± 0.003 | 115.866 | ＜0.01 |
| *Pseudoxanthobacter* | 0.05 ± 0.03 | Not observed | 110.691 | ＜0.01 |
| *Rhodococcus* | 0.23 ± 0.13 | 0.02 ± 0.03 | 105.121 | ＜0.01 |
| *Reyranella* | 0.11 ± 0.06 | 0.02 ± 0.02 | 100.129 | ＜0.01 |
| *Iamia* | 0.05 ± 0.03 | Not observed | 98.342 | ＜0.01 |
| *Luteimonas* | 0.15 ± 0.10 | Not observed | 97.387 | ＜0.01 |
| *ZOR0006* | 0.03 ± 0.02 | Not observed | 96.523 | ＜0.01 |
| *alphaI_cluster* | 0.03 ± 0.02 | Not observed | 62.202 | ＜0.01 |
| *Legionella* | 0.04 ± 0.03 | 0.01 ± 0.01 | 60.749 | ＜0.01 |
| *Leifsonia* | 0.09 ± 0.04 | 0.03 ± 0.04 | 51.080 | ＜0.01 |
| *Luteolibacter* | 0.06 ± 0.06 | Not observed | 40.078 | ＜0.01 |
| *Aeromonas* | 0.07 ± 0.04 | 0.02 ± 0.05 | 32.273 | ＜0.01 |
| *Pseudoxanthomonas* | 0.13 ± 0.16 | Not observed | 31.313 | ＜0.01 |
| *Actinomyces* | 0.02 ± 0.02 | Not observed | 28.960 | ＜0.01 |
| *Bacteroides* | 0.05 ± 0.06 | Not observed | 27.809 | ＜0.01 |
| *Pelomonas* | 0.02 ± 0.02 | Not observed | 25.469 | ＜0.01 |
| *Neisseria* | 0.02 ± 0.03 | Not observed | 22.233 | ＜0.01 |
| *Flavobacterium* | 0.02 ± 0.02 | 0.01 ± 0.01 | 17.925 | ＜0.01 |
| *Cellvibrio* | 0.02 ± 0.03 | Not observed | 16.629 | ＜0.01 |
| *Thermomonas* | 0.01 ± 0.03 | Not observed | 8.014 | ＜0.01 |
| *Fusobacterium* | 0.01 ± 0.02 | Not observed | 6.274 | ＜0.05 |
| *Candidatus_Berkiella* | 0.01 ± 0.03 | Not observed | 4.507 | ＜0.05 |
| *Terrisporobacter* | Not observed | 0.04 ± 0.05 | 36.131 | ＜0.01 |
| *Clostridium_sensu_stricto_1* | 0.07 ± 0.01 | 0.29 ± 0.34 | 34.063 | ＜0.01 |
| *Edwardsiella* | ＜0.01 | 0.05 ± 0.06 | 24.602 | ＜0.01 |
| *Peptostreptococcaceae* | Not observed | 0.15 ± 0.21 | 23.128 | ＜0.01 |
| *Plesiomonas* | 0.02 ± 0.02 | 0.17 ± 0.21 | 22.161 | ＜0.01 |
| *Epulopiscium* | Not observed | 0.04 ± 0.06 | 17.308 | ＜0.01 |
| *Mycoplasma* | ＜0.01 | 0.23 ± 0.42 | 14.760 | ＜0.01 |
| *Alsobacter* | 0.03 ± 0.03 | 0.10 ± 0.15 | 10.414 | ＜0.01 |
