# Supplementary material for: Gut microbiota of two invasive fishes respond differently to temperature
Source: Front Microbiol. 2023 Mar 28;14:1087777. doi: 10.3389/fmicb.2023.1087777 (PMC10088563; doi:10.3389/fmicb.2023.1087777)
Supplement: Supplementary file 3 [file Table_2.DOCX]

**Table S2**

Results of PERMANOVA models based on distance matrices calculated using Bray-Curtis dissimilarity, assessing the effects of temperature and time on gut bacterial community composition of Common carps and Largemouth basses. The F statistic for each variable is presented, along with FDR corrected p-values (q-values).

|  | **Metric** | **Factor** | **F statistic** | **q-value** |
| --- | --- | --- | --- | --- |
| **Common carp** | **bray-curtis** |  |  |  |
|  |  | temp | 3.882 | 0.013 |
|  |  | time | 16.900 | 0.001 |
| **Largemouth bass** | **bray-curtis** |  |  |  |
|  |  | temp | 3.368 | 0.042 |
|  |  | time | 2.231 | 0.074 |
