# Supplementary material for: Gut microbiota of two invasive fishes respond differently to temperature
Source: Front Microbiol. 2023 Mar 28;14:1087777. doi: 10.3389/fmicb.2023.1087777 (PMC10088563; doi:10.3389/fmicb.2023.1087777)
Supplement: Supplementary file 4 [file Table_3.docx]

**TABLE** **S3** Results of pairwise PERMANOVA models between two temperature treatments within each of three experimental time points in common carps and largemouth basses gut microbiota based on three metrics of community dissimilarity (Bray-Curtis, unweighted UniFrac, and weighted UniFrac). The F statistic for temperature within each model is presented, along with FDR corrected p-values (q-values).

|  | **Metric** | **Time point**  **(hours)** | **F statistic** | **q-value** |
| --- | --- | --- | --- | --- |
| **Common carp** | **bray-curtis** |  |  |  |
|  |  | 24 | 1.582 | 0.201 |
|  |  | 72 | 2.219 | 0.075 |
|  |  | 168 | 4.483 | 0.001 |
|  | **unweighted unifrac** |  |  |  |
|  |  | 24 | 2.222 | 0.007 |
|  |  | 72 | 2.572 | 0.002 |
|  |  | 168 | 2.678 | 0.001 |
|  | **weighted unifrac** |  |  |  |
|  |  | 24 | 1.112 | 0.307 |
|  |  | 72 | 1.640 | 0.179 |
|  |  | 168 | 3.805 | 0.001 |
| **Largemouth bass** | **bray-curtis** |  |  |  |
|  |  | 24 | 2.011 | 0.109 |
|  |  | 72 | 2.877 | 0.025 |
|  |  | 168 | 0.505 | 0.737 |
|  | **unweighted unifrac** |  |  |  |
|  |  | 24 | 1.680 | 0.097 |
|  |  | 72 | 3.142 | 0.025 |
|  |  | 168 | 0.928 | 0.412 |
|  | **weighted unifrac** |  |  |  |
|  |  | 24 | 1.999 | 0.159 |
|  |  | 72 | 1.941 | 0.123 |
|  |  | 168 | 0.445 | 0.743 |
