# Supplementary material for: Gut microbiota of two invasive fishes respond differently to temperature
Source: Front Microbiol. 2023 Mar 28;14:1087777. doi: 10.3389/fmicb.2023.1087777 (PMC10088563; doi:10.3389/fmicb.2023.1087777)
Supplement: Supplementary file 5 [file Table_4.DOCX]

**TABLE S4** Bacterial phyla and genera in common carp and largemouth bass gut microbiota which had relative abundances that were significantly affected by time. The symbol in the time column indicates the direction of the relationship of that taxa’s abundance with time (adding or subtracting). Significance was determined with ANCOVA models and the F statistic for each variable is presented, along with FDR corrected p-values (q-values). Taxa are ordered by their effect size.

| **Common carp** | **Phylm** | **Time** | **F statistic** | **p value** |
| --- | --- | --- | --- | --- |
|  | Planctomycetota | + | 8.932 | ＜0.001 |
|  | Actinobacteriota | + | 7.811 | ＜0.001 |
|  | Proteobacteria | + | 6.237 | ＜0.001 |
|  | Verrucomicrobiota | - | 4.839 | ＜0.05 |
|  | Firmicutes | + | 2.759 | ＜0.05 |
|  | Desulfobacterota | + | 2.031 | ＜0.05 |
|  | **Genera** |  |  |  |
|  | *Legionella* | - | 24.360 | ＜0.001 |
|  | *Pseudoxanthomonas* | + | 15.786 | ＜0.001 |
|  | *Pseudoxanthobacter* | - | 12.348 | ＜0.001 |
|  | *Candidatus_Berkiella* | + | 11.237 | ＜0.001 |
|  | *alphaI_cluster* | + | 10.277 | ＜0.001 |
|  | *Cellvibrio* | + | 8.155 | ＜0.001 |
|  | *Cetobacterium* | - | 7.992 | ＜0.001 |
|  | *Mycobacterium* | - | 7.945 | ＜0.001 |
|  | *Rhodobacter* | + | 7.695 | ＜0.001 |
|  | *Rhodococcus* | + | 6.626 | ＜0.001 |
|  | *Kaistia* | + | 5.914 | ＜0.001 |
|  | *Plesiomonas* | - | 5.756 | ＜0.01 |
|  | *Gemmobacter* | + | 5.450 | ＜0.01 |
|  | *Acinetobacter* | + | 5.394 | ＜0.001 |
|  | *Aurantimicrobium* | + | 4.906 | ＜0.05 |
|  | *Macellibacteroides* | - | 4.711 | ＜0.001 |
|  | *Luteimonas* | + | 4.404 | ＜0.001 |
|  | *Microbacterium* | + | 4.318 | ＜0.05 |
|  | *Thermomonas* | + | 4.205 | ＜0.05 |
|  | *ZOR0006* | + | 4.058 | ＜0.01 |
|  | *IMCC26207* | - | 3.945 | ＜0.001 |
|  | *Clostridium_sensu_stricto_1* | - | 3.796 | ＜0.01 |
|  | *Pelomonas* | + | 3.786 | ＜0.01 |
|  | *Deinococcus* | + | 3.704 | ＜0.05 |
|  | *Aminobacter* | + | 3.668 | ＜0.01 |
|  | *Phreatobacter* | + | 3.565 | ＜0.01 |
|  | *Bacteroides* | - | 2.931 | ＜0.05 |
|  | *Reyranella* | - | 2.861 | ＜0.05 |
|  | *Flavobacterium* | + | 2.446 | ＜0.05 |
| **Largemouth bass** | **Phylm** |  |  |  |
|  | Proteobacteria | + | 2.659 | ＜0.05 |
|  | **Genera** |  |  |  |
|  | *Edwardsiella* | + | 4.725 | ＜0.01 |
|  | *Luteolibacter* | + | 3.633 | ＜0.01 |
|  | *Alsobacter* | + | 2.938 | ＜0.05 |
|  | *Rhodoluna* | + | 2.926 | ＜0.05 |
|  | *Pelomonas* | + | 2.707 | ＜0.05 |
