# Supplementary figures and images for: Gut microbiota of two invasive fishes respond differently to temperature
Source: Front Microbiol. 2023 Mar 28;14:1087777. doi: 10.3389/fmicb.2023.1087777 (PMC10088563; doi:10.3389/fmicb.2023.1087777)

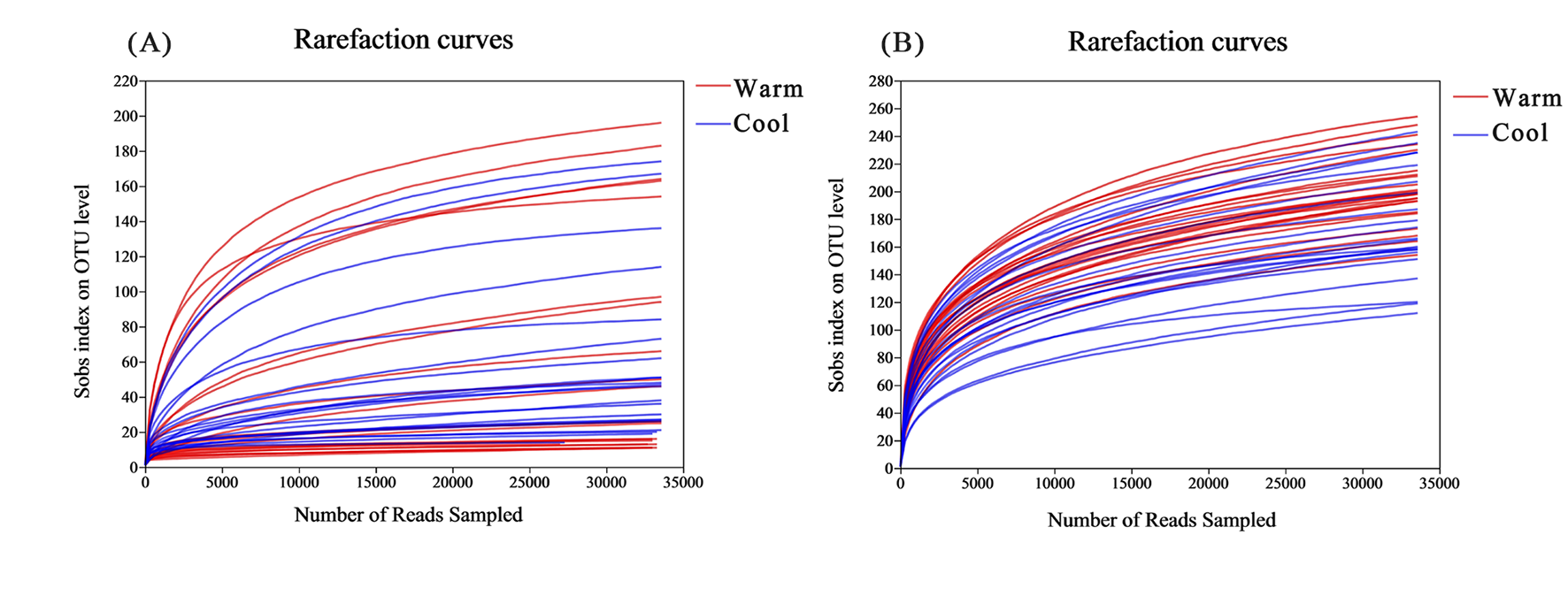

Supplement: Supplementary Figure 1 — Rarefaction curves of intestinal microbial samples based on Illumina MiSeq sequencing. (A) Common carp. (B) Largemouth bass. [file Image_1.TIF]

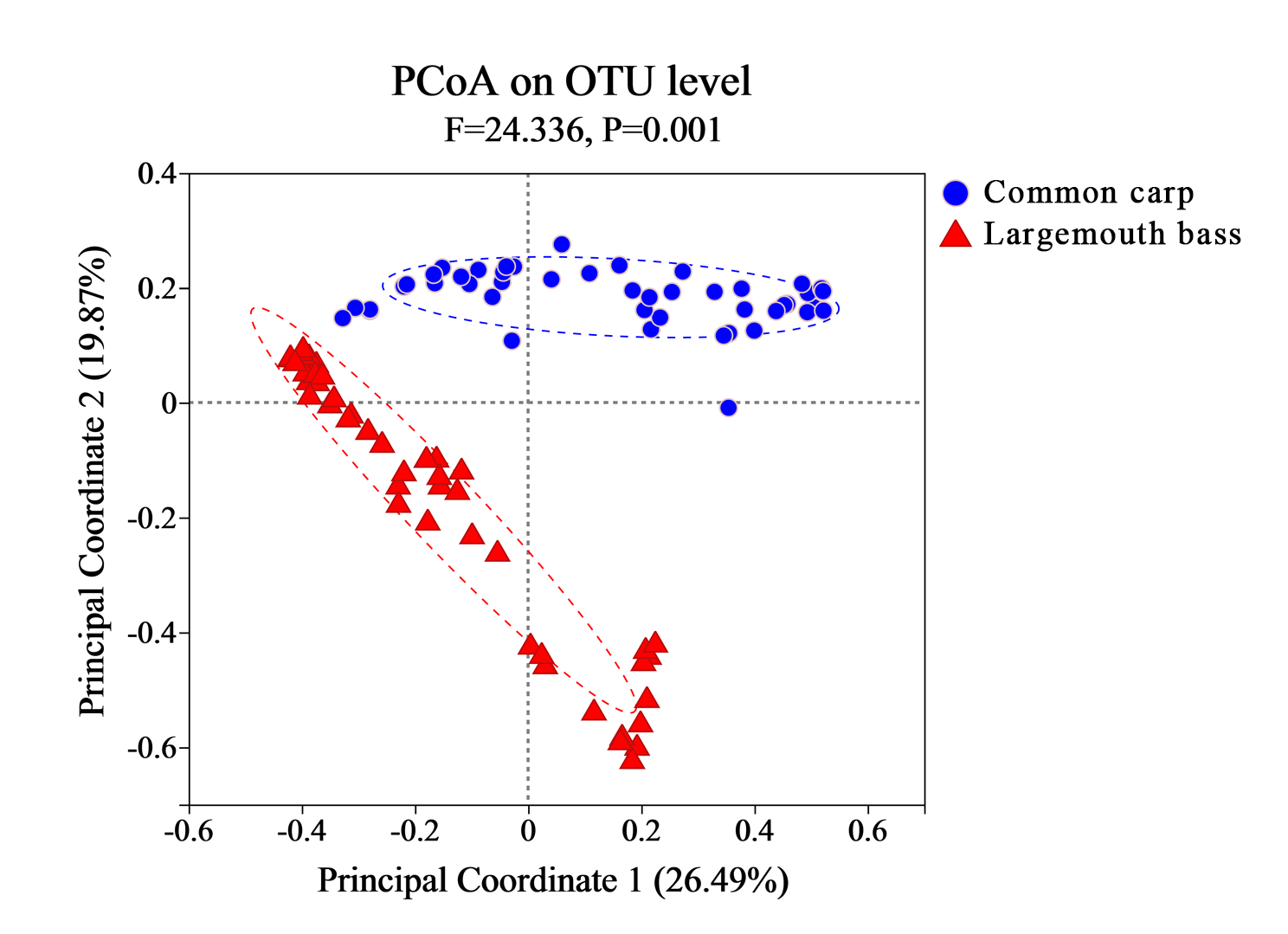

Supplement: Supplementary Figure 2 — Principal coordinate analysis plot displaying gut microbiota of common carp and largemouth bass, based on Bray–Curtis dissimilarity across samples. The plot includes all samples, from each temperature and time point. Percentages on PCoA axes represent the proportion of variation explained by each axis. Two groups are significantly different from one another (PERMANOVA, FDR P = 0.001). [file Image_2.TIF]

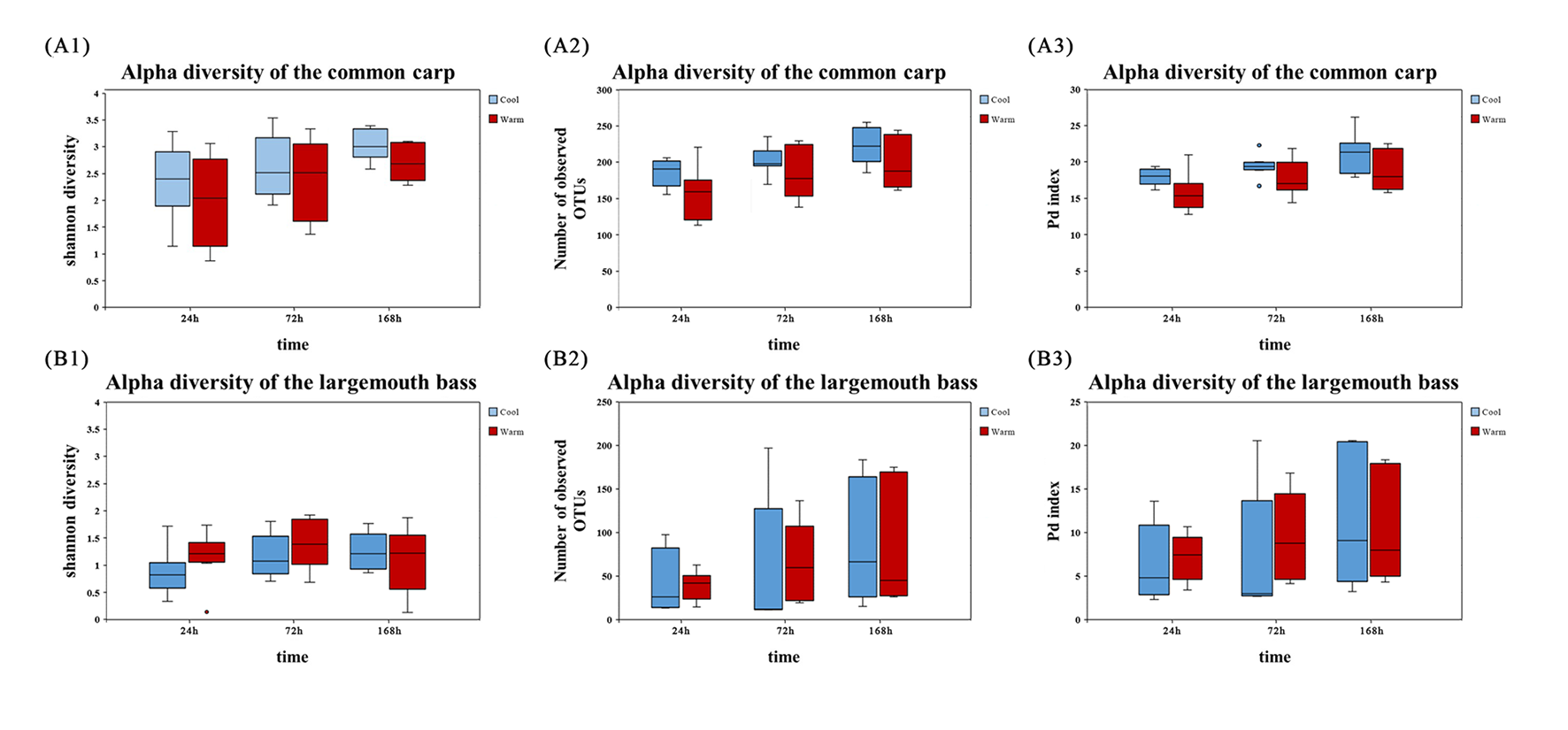

Supplement: Supplementary Figure 3 — Alpha diversity of gut microbiota of fishes in two temperature treatments at three time points. The center bolded line represents the median, the length of the box represents the IQR, and the whiskers extend to 1.5xIQR. Points beyond this value are plotted individually. Common carp (A1–A3), Shannon diversity, OUTs and PD index of common carp was significantly impacted by time (GLMM, p < 0.001), but not by temperature (GLMM, p > 0.05). Largemouth bass (B1–B3), OUTs and PD index of largemouth bass was significantly impacted by time (GLMM, p < 0.05), but not by temperature (GLMM, p > 0.05). [file Image_3.TIF]

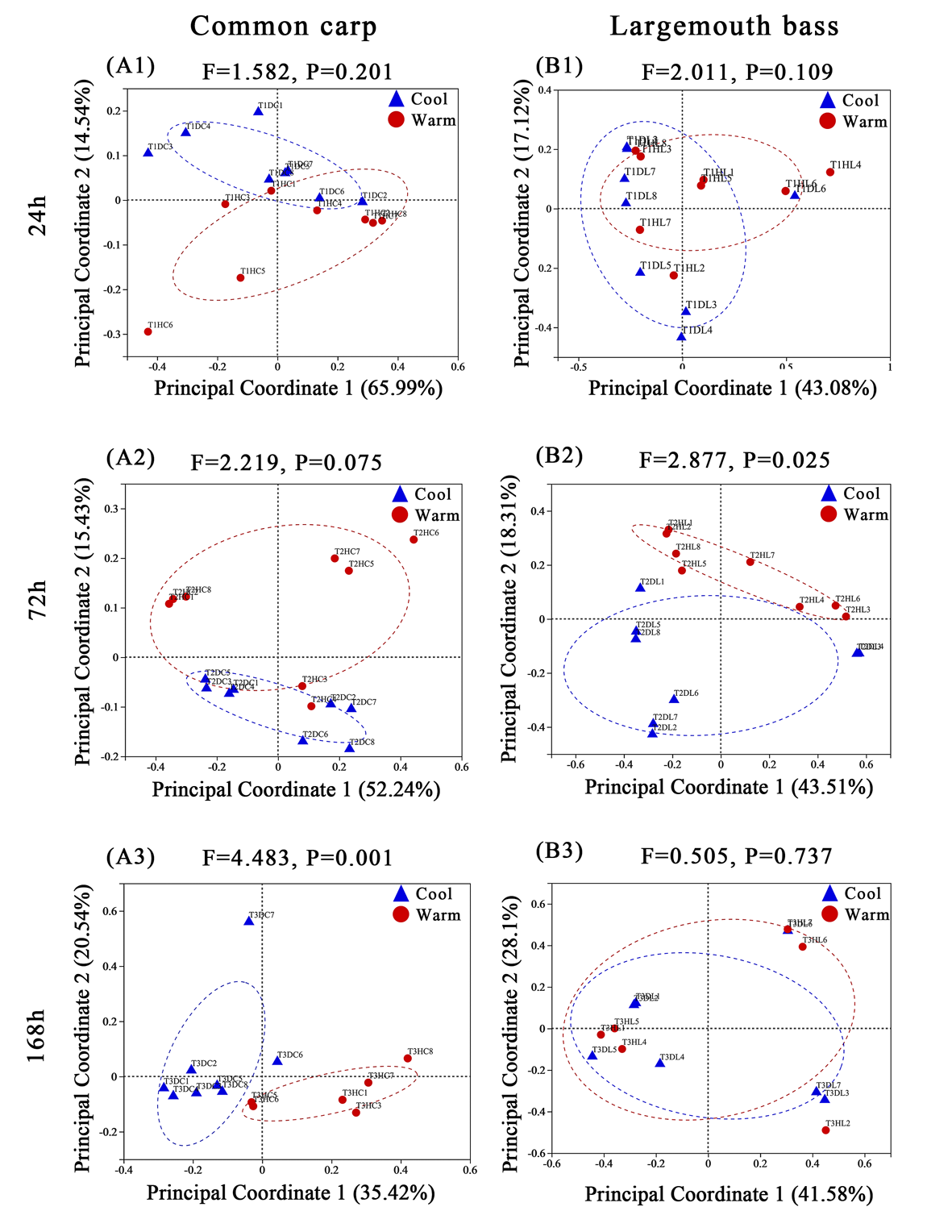

Supplement: Supplementary Figure 4 — Principal coordinate analyses plots of common carp and largemouth bass gut microbiota at the 24 h (A1,B1), 72 h (A2,B2), and 168 h (A3,B3) experimental time points, based on Bray–Curtis dissimilarity across samples. Points are colored by temperature treatment (red = warm; blue = cool), and ellipses represent the 95% confidence interval of that treatment group. On each plot, the results of PERMANOVA models assessing the temperature effects on gut microbial community composition at that time point are displayed, including the F statistic from the model, and the FDR corrected p-values (q-value). Percentages on the axes of PCoA plots indicate the proportion of variation explained by that axis. [file Image_4.TIF]

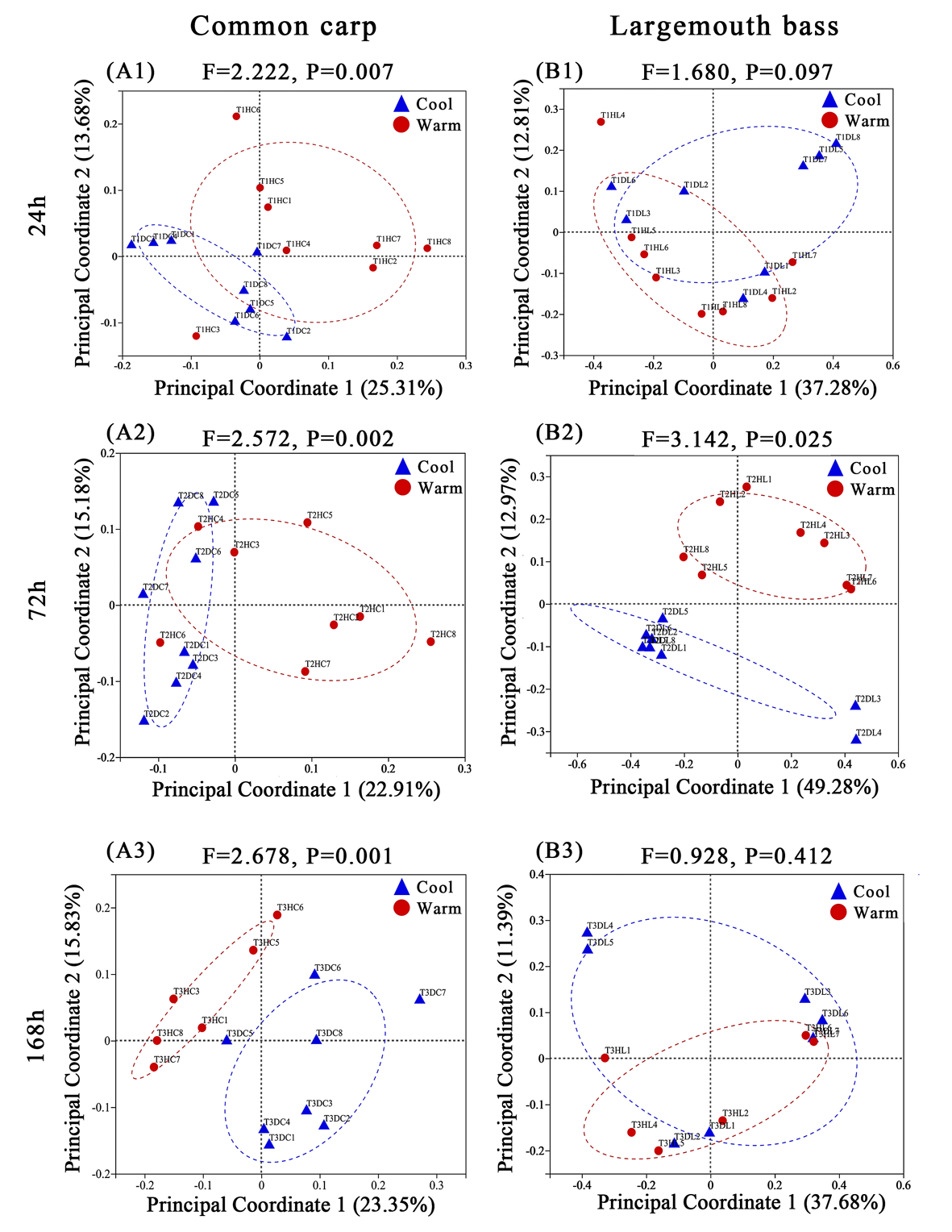

Supplement: Supplementary Figure 5 — Principal coordinate analyses plots of common carp and largemouth bass gut microbiota at the 24 h (A1,B1), 72 h (A2,B2), and 168 h (A3,B3) experimental time points, based on unweighted UniFrac distance across samples. Points are colored by temperature treatment (red = warm; blue = cool), and ellipses represent the 95% confidence interval of that treatment group. On each plot, the results of PERMANOVA models assessing the temperature effects on gut microbial community composition at that time point are displayed, including the F statistic from the model, and the FDR corrected p-values (q-value). Percentages on the axes of PCoA plots indicate the proportion of variation explained by that axis. [file Image_5.TIF]
